# Subchondral Bone Plate Thickening Precedes Chondrocyte Apoptosis and Cartilage Degradation in Spontaneous Animal Models of Osteoarthritis

**DOI:** 10.1155/2014/606870

**Published:** 2014-06-18

**Authors:** Zaitunnatakhin Zamli, Kate Robson Brown, John F. Tarlton, Mike A. Adams, Georgina E. Torlot, Charlie Cartwright, William A. Cook, Kristiina Vassilevskaja, Mohammed Sharif

**Affiliations:** ^1^Centre for Comparative and Clinical Anatomy, University of Bristol, Southwell Street, Bristol BS2 8EJ, UK; ^2^Imaging Laboratory, Department of Archaeology and Anthropology, University of Bristol, Bristol BS8 1UU, UK; ^3^School of Veterinary Science, University of Bristol, Langford, Bristol BS40 5DU, UK; ^4^School of Clinical Sciences, University of Bristol, Musculoskeletal Research Unit, Avon Orthopaedic Centre, Southmead Hospital, Bristol BS10 5NB, UK

## Abstract

Osteoarthritis (OA) is the most common joint disorder characterised by bone remodelling and cartilage degradation and associated with chondrocyte apoptosis. These processes were investigated at 10, 16, 24, and 30 weeks in Dunkin Hartley (DH) and Bristol Strain 2 (BS2) guinea pigs that develop OA spontaneously. Both strains had a more pronounced chondrocyte apoptosis, cartilage degradation, and subchondral bone changes in the medial than the lateral side of the tibia, and between strains, the changes were always greater and faster in DH than BS2. In the medial side, a significant increase of chondrocyte apoptosis and cartilage degradation was observed in DH between 24 and 30 weeks of age preceded by a progressive thickening and stiffening of subchondral bone plate (Sbp). The Sbp thickness consistently increased over the 30-week study period but the bone mineral density (BMD) of the Sbp gradually decreased after 16 weeks. The absence of these changes in the medial side of BS2 may indicate that the Sbp of DH was undergoing remodelling. Chondrocyte apoptosis was largely confined to the deep zone of articular cartilage and correlated with thickness of the subchondral bone plate suggesting that cartilage degradation and chondrocyte apoptosis may be a consequence of continuous bone remodelling during the development of OA in these animal models of OA.

## 1. Introduction

Osteoarthritis (OA) is a common degenerative joint disorder that leads to pain and immobility, especially in elderly populations. Progression of spontaneous OA is typically slow and usually diagnosed at later stage of the disorder. The aetiology of OA is complex and multifactorial but the established OA joint always exhibits the common feature of cartilage degradation. Moreover, the incidence of OA in humans or animals is associated with abnormal subchondral bone remodelling [1, 2]. Thus studies have shown that increased subchondral bone turnover is accompanied by structural changes of the bone which include increased thickness of subchondral bone plate (Sbp) and trabecular bone (Tb) [3–5] and formation of subchondral bone cysts and peripheral osteophytes [4, 6–8], as evidenced by radiographic and morphometric analysis of the osteoarthritic joint. Other studies have demonstrated that there is an increase of BMD [1, 9, 10] and bone mineral content (BMC) [11] in OA. Although the above findings support the importance of subchondral bone changes in the pathogenesis of OA, the hypothesis that these changes initiate idiopathic OA is still unproven. Moreover, the role of subchondral bone in relation to chondrocyte apoptosis and cartilage degradation has yet to be established.

Chondrocyte apoptosis has been shown to be involved in the etiopathogenesis of OA. Studies of human and animal OA have demonstrated that chondrocyte death by apoptosis is at least partly responsible for cellularity reduction, cartilage breakdown, and matrix turnover abnormalities [12–18]. Moreover, the localisation of chondrocyte apoptosis near the lesion area of osteoarthritic cartilage further emphasises the importance of chondrocyte death in the progression of OA [12, 13]. Recently, the role of chondrocyte apoptosis in the initiation and progression of OA has received a great deal of attention [19] but the relationship between chondrocyte apoptosis and subchondral bone change has received little or no attention and needs to be addressed, particularly as osteoblasts from OA subchondral bone are known to influence the survivability of chondrocyte in* in vitro *studies of human tissues [20, 21]. Accordingly, the aims of the current study are to determine the sequence and the relationship between subchondral bone changes, chondrocyte apoptosis, and cartilage degradation, during the development of OA in two spontaneous animal models of OA, the rapidly developing DH guinea pig OA, and the delayed OA seen in the BS2 strain.

## 2. Materials and Methods

### 2.1. Animals

Male Dunkin Hartley (DH) guinea pigs were purchased from Harlan (Harlan Laboratories Ltd., UK) and Bristol Strain 2 (BS2) guinea pigs were inbred at the Animal Services Unit of University of Bristol. The animals were reared in an environmentally control room (12 h-lighting/22°C, 1-2 animals/cage) and had a free access to food (Harlan Teklad, UK) and water (contained 1-2 mg/mL of vitamin C).

### 2.2. Sample Collection

Six animals from each strain were euthanized at 10, 16, 24, and 30 weeks of age by an overdose of euthatal via intraperitoneal injection. The right knee joints were dislocated and the tibias were cleaned of soft tissues. All the above procedures were approved by the Research Ethics Committee, University of Bristol (Win number: UB/10/024).

### 2.3. Microcomputed Tomography Scanning (Micro-CT)

The proximal part of the tibia was scanned using a micro-CT scanner (Bruker SkyScan 1172, Kontich, Belgium) at 180° rotation with a resolution of 14.7 *μ*m in AP position. The voltage of the X-ray tube was set to 65 kV and the current was 153 *μ*A with a 0.5 mm aluminium filter. The image reconstruction, using a modified Feldkamp algorithm, was undertaken in the software program NRecon (Version 1.6.1.5, Skyscan, Kontich, Belgium) and images were saved in BMP format. The data were analysed using Bruker SkyScan CT-Analyser software (Version 1.9.2.5, Skyscan, Kontich, Belgium).

### 2.4. Subchondral Bone Plate Thickness (SbpTh)

A set of reconstructed images of each sample was opened in CTAn program. By using the multiple reslice model option, three nonconsecutive frontal sections were made in the middle of the tibial plateau. A standard grid was superimposed on each section so that the SbpTh were measured at the same regions across the samples. With the grid as a guide, vertical lines were drawn from the osteochondral junction down to marrow space on the medial/lateral side of the tibial plateau.

### 2.5. Trabecular Bone (Tb) Morphometry Analysis

A cylindrical volume of interest (VOI) (diameter: 2 mm, height: 0.36 mm) was placed in the middle of the condyles on each side of the tibial epiphysis. Anatomical landmarks were used as a guide to ensure that the VOI was positioned in such a way that the subchondral bone plate (Sbp) and the growth plate were excluded. A number of Tb structural parameters were selected for a 3D analysis of the VOI. These parameters included the bone volume/tissue volume (BV/TV), trabecular thickness (Tb.Th), trabecular number (Tb.N), trabecular separation (Tb.Sp), and trabecular pattern factor (Tb.Pf).

### 2.6. BMD Measurement Using a Micro-CT

Two densitometric phantoms of known BMD (0.25 and 0.75 g/cm^3^ of calcium hydroxyapatite) were scanned in similar condition to the tibia. Attenuation coefficient value of each phantom was recorded and used for BMD calibration process. The BMD of Sbp and Tb was measured in the middle of medial/lateral tibial plateau (VOI: diameter: 2 mm, height: 0.15 mm) and in the same region as described in the Tb morphometry analysis, respectively.

### 2.7. Articular Cartilage (AC) Degradation and Chondrocyte Apoptosis

Histological sections for microscopic scoring (degree of cartilage degradation) and caspase-3 staining were prepared as previously described [22]. Briefly, the proximal part of the right tibial epiphysis was snap-frozen in cold isopentane for 3 minutes and embedded in SCEM (Section Lab, Japan). The embedded samples were sectioned at 7 *μ*m according to Kawamoto's film method [23]. The sections were then stained and scored three times in accordance with a modified Osteoarthritis Research Society International (OARSI) recommended scoring scheme for cartilage degradation in guinea pigs [24].

Chondrocyte apoptosis was identified by immunohistochemical staining of caspase-3 expression [14]. Briefly, the sections were fixed in 4% formalin and blocked using a goat serum (1 : 5, 1 hour, Vector). The sections were then incubated with primary antibody against active caspase-3 (1 : 200, 1 h, R&D systems) and followed by biotin-labelled secondary antibody (1 : 200, 1 hour, a goat anti-rabbit antibody, Millipore). Extra-avidin alkaline phosphatase conjugate (1 : 100, Sigma Aldrich, UK) was added and left to incubate for 30 minutes. Next, the sections were incubated with fast red substrates (Sigma Aldrich, UK) for 10 minutes and counterstained with Carazzi's haematoxylin (Section Lab, Japan) for 2 minutes. The percentage of chondrocytes apoptosis was calculated by dividing the number of positive staining chondrocytes in the medial/lateral side of AC with the total number of chondrocytes in the respective side and multiplied by 100.

### 2.8. Statistical Analysis

Comparisons were made between strains and also between the medial and lateral side of each strain to account for the effect of growth. Statistical analysis was performed using PASW Statistic 18 software. A Kolmogorov-Smirnov test was used to test the normality of data distribution. The data were analysed by comparing between the medial and lateral sides of the tibia, strains, and time points. Correlations between cartilage and subchondral bone parameters were also performed. The data were presented as mean ± SEM and a significant difference of *P* < 0.05 and *P* < 0.01 was denoted as ∗ and ∗∗, respectively.

## 3. Results

### 3.1. Subchondral Bone Plate (Sbp) Changes in OA

**Figure 1:**
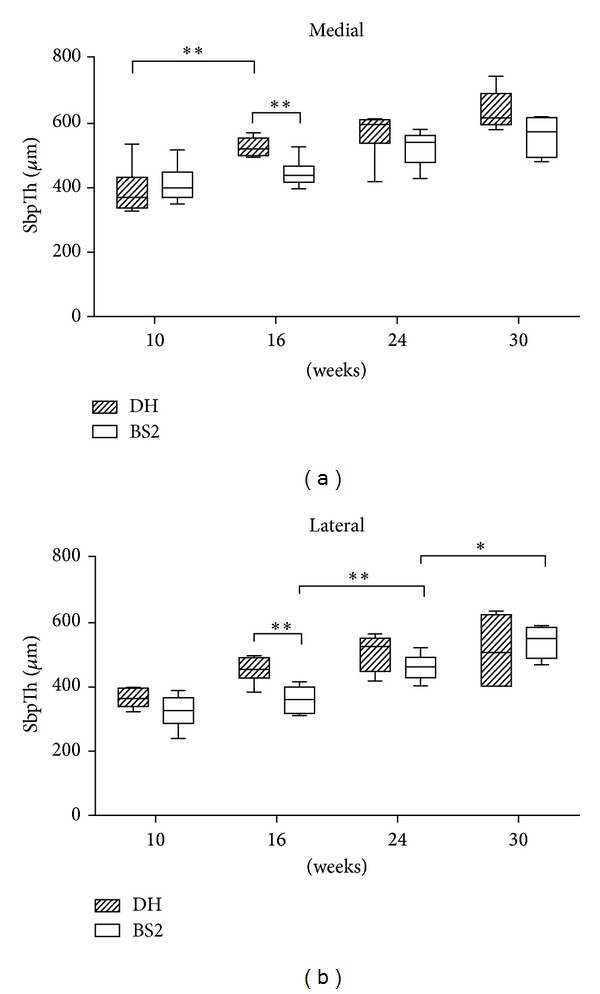
SbpTh of the medial and the lateral side of DH and BS2 at four time points. SbpTh of DH (*n* = 6) was significantly higher than BS2 (*n* = 6) at 16 weeks, both in the medial and lateral side (unpaired *t*-test, *P* < 0.01). A significant progression of Sbp thickening was observed in the medial side of DH (one-way ANOVA followed by Bonferroni post hoc test, *P* < 0.01) and the lateral side of BS2 (one-way ANOVA followed by Bonferroni post hoc test, between 16 and 24 weeks: *P* < 0.01, between 24 and 30 weeks: *P* < 0.05). The data are expressed as mean ± SEM.

An age-related increase in Sbp thickness (SbpTh) was observed, particularly in the medial side of tibial condyle (Figure 1). SbpTh was greater in DH than BS2 at most time points but statistically significant only at 16 weeks of age (at the medial side, DH: 526.6 *μ*m ± 11.8, BS2: 445.4 *μ*m ± 18.0, *P* < 0.01; at lateral side, DH: 453.8 *μ*m ± 16.8, BS2: 360.9 *μ*m ± 16.8, *P* < 0.01). In the medial side, a significant progression of SbpTh was found in DH between 10 and 16 weeks (*P* < 0.01). In contrast, BS2 demonstrated no significant change in SbpTh medially and in fact showed an increase of SbpTh in the lateral side between 16 and 30 weeks of age (between 16 and 24 weeks: *P* < 0.01; between 24 and 30 weeks: *P* < 0.05).

**Figure 2:**
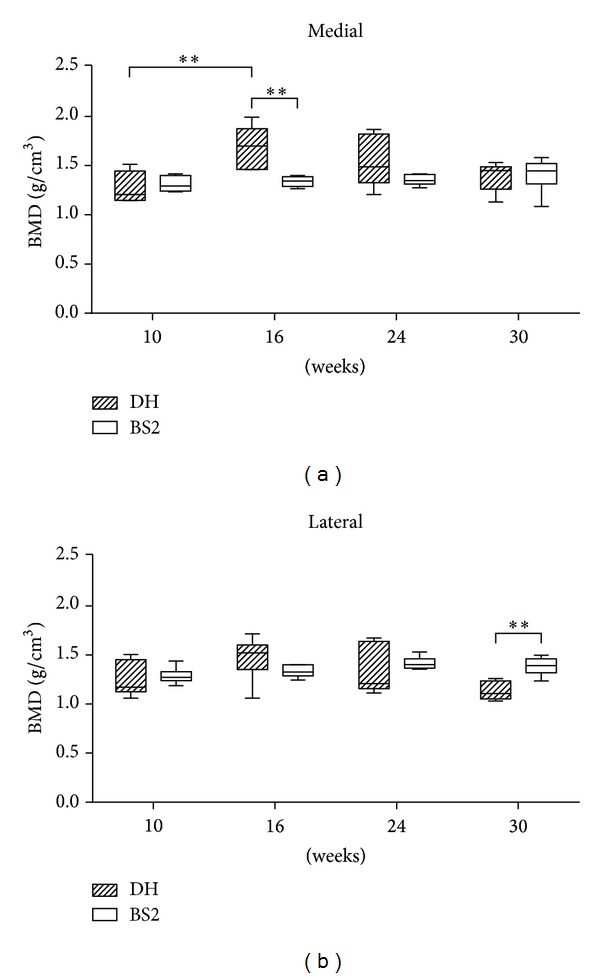
BMD of Sbp measured by a micro-CT scanner. DH (*n* = 6) had a significantly denser medial Sbp than BS2 (*n* = 6) at 16 weeks of age (unpaired *t*-test, *P* < 0.01). However, at 30 weeks, the opposite was observed in the lateral side of Sbp (unpaired *t*-test, *P* < 0.01). Over time, only the medial side of DH showed a dramatic increase of BMD between 10 and 16 weeks (one-way ANOVA followed by Bonferroni post hoc test, *P* < 0.01). The data are expressed as mean ± SEM.

Figure 2 shows that BMD in the medial side of DH Sbp (1.7 g/cm^3^ ± 0.1) was significantly higher than BS2 (1.3 g/cm^3^ ± 0.02) at 16 weeks of age (*P* < 0.01). However, at 30 weeks, the reverse was noticed in the lateral side of Sbp (DH: 1.1 g/cm^3^ ± 0.04, BS2: 1.4 g/cm^3^ ± 0.04, *P* < 0.01). In contrast to the findings for SbpTh, the BMD of BS2 shows a gradual increase over the study period. However, in the DH strain an initial increase in BMD was followed by a decrease, and this initial increase between 10 and 16 weeks was only significant in the medial (*P* < 0.01) but not in the lateral side.

### 3.2. Trabecular Bone (Tb) Changes in OA

**Figure 3:**
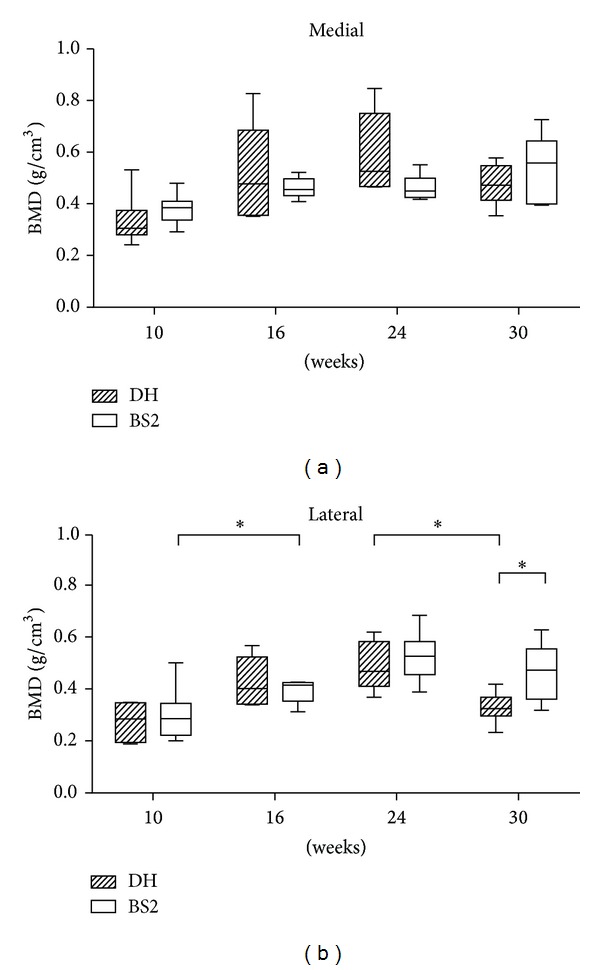
BMD of the medial and lateral Tb of DH (*n* = 6) and BS2 (*n* = 6) was measured using a micro-CT scanner at four time points. The data are expressed as mean ± SEM and statistically tested between strains and time points using unpaired *t*-test and one-way ANOVA (followed by Bonferroni post hoc test), respectively. A significant difference of *P* < 0.01 and *P* < 0.05 was denoted as ∗∗ and ∗, respectively.

Figure 3 illustrates the BMD changes in Tb as measured using microcomputed tomography. The mean of BMD was greater in the medial than the lateral side of Tb in both strains. It peaked at 24 weeks (at the medial side, DH: 0.592 g/cm^3^ ± 0.06; at the lateral side, DH: 0.487 g/cm^3^ ± 0.04; BS2: 0.525 g/cm^3^ ± 0.04) and thereafter decreased at the final time point in all tibias except in the medial side of BS2. Furthermore, over time, only the lateral side of Tb showed a dramatic increase and decrease of BMD in BS2 (between 10 and 16 weeks, *P* < 0.05) and DH (between 24 and 30 weeks, *P* < 0.05), respectively.

**Figure 4:**
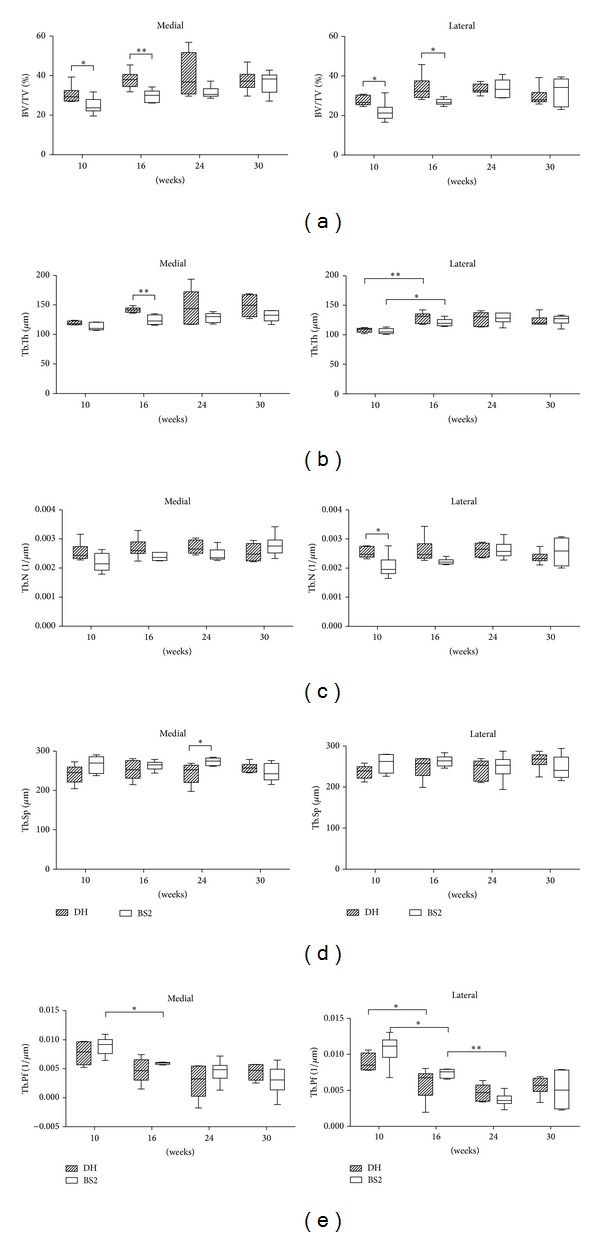
Tb morphometry of the medial and lateral side of DH and BS2. (a) BV/TV (%), (b) Tb.Th (*μ*m), (c) Tb.N (1/*μ*m), (d) Tb.Sp (*μ*m), and (e) Tb.Pf (1/*μ*m) were measured using a micro-CT scanner at four time points (*n* = 6 per strain at each time point). The data are expressed as mean ± SEM and statistically tested between strains and time points using unpaired *t*-test and one-way ANOVA (followed by Bonferroni post hoc test), respectively. A significant difference of *P* < 0.01 and *P* < 0.05 was denoted as ∗∗ and ∗, respectively.

The morphometric changes of Tb in the medial and lateral side of DH/BS2 are shown in Figure 4. Data from the bone volume per tissue volume (BV/TV) demonstrated that DH had significantly higher Tb volume fraction than BS2 at 10 weeks (at medial side, DH: 30.4% ± 1.9, BS2: 24.8% ± 1.7, *P* < 0.05; at lateral side, DH: 27.4% ± 1.0, BS2: 21.9% ± 2.1, *P* < 0.05) and at 16 weeks of age (at medial side, DH: 37.9% ± 1.8, BS2: 29.8% ± 1.3, *P* < 0.01; at lateral side, DH: 33.7% ± 2.6, BS2: 26.8% ± 0.7, *P* < 0.05). Over time, the BV/TV increased first then decreased in all tibias, except in the medial side of BS2 where it continued to increase at 30 weeks of age (Figure 4(a)).

The medial side of DH showed a gradual increase in trabecular thickness (Tb.Th) with age and was greater at all time points than the BS2 but only significant at 16 weeks (DH: 141.7 *μ*m ± 1.9, BS2: 124.5 *μ*m ± 3.2, *P* < 0.01). This increase was more marked medially than laterally. In the lateral side both strains showed a marked increase in Tb.Th between 10 and 16 weeks of age (DH: *P* < 0.01, BS2: *P* < 0.05), before decreasing at the later time points (Figure 4(b)).

Trabecular number (Tb.N) did not show any appreciable difference between sides or strains other than a marginally greater Tb.N in the DH at early time points but only significant in the medial at 10 weeks (DH: 0.003/*μ*m, BS2: 0.002/*μ*m, *P* < 0.05). The Tb.N showed an increase up to 24 weeks before decreasing at the final time point. This trend was true for both strains, excluding the medial side of BS2 (Figure 4(c)).

DH had a smaller trabecular separation (Tb.Sp) than BS2 for the first 24 weeks of age. This was significant in the medial side at 24 weeks (DH: 243.6 *μ*m ± 10.9, BS2: 273.5 *μ*m ± 4.1, *P* < 0.05). However, at the 30 weeks, the reverse was observed, though not significant (medial side, DH: 257.8 *μ*m ± 5.2, BS2: 245.6 *μ*m ± 9.6, *P* > 0.05; lateral side, DH: 264.9 *μ*m ± 8.7, BS2: 247.3 *μ*m ± 11.6, *P* > 0.05) (Figure 4(d)).

Trabecular pattern factor (Tb.Pf), a measure of trabecular connectivity, was lower in the DH at the earlier time points but lower in the BS2 at later time points, though not significant. In the medial side of BS, Tb.Pf decreased linearly with age and was statistically significant between 10 and 16 weeks (*P* < 0.05). In contrast, Tb.Pf in DH and the lateral side of BS2 decreased up to 24 weeks and then increased at the end of study period. A significant decrease was observed in Tb.Pf in the lateral side of DH and BS2 up to 16 (*P* < 0.05) and 24 weeks (*P* < 0.05), respectively (Figure 4(e)).

### 3.3. Body Weight and Microscopic Changes in DH and BS2

The body weight of DH was about 19% (*P* < 0.01) higher than BS2 (Table 1). However, both strains had a similar rate of growth and rapid weight gain up to 24 weeks (DH: 1083.3 g ± 31.8; BS2: 885.8 g ± 32.5) and fairly constant weight gain between 24 and 30 weeks. With aging, the progression of cartilage degradation was also seen especially in the medial side of AC (Table 1). It was greater in DH than BS2 but was statistically significant only at 30 weeks of age (DH: 16.0 ± 0.8, BS2: 12.5 ± 1.2, *P* < 0.05). Progression of cartilage damage was noted only between 24 and 30 weeks in the medial side of DH (*P* < 0.01).

### 3.4. Relation between Chondrocyte Apoptosis and Subchondral Bone and AC Changes

Chondrocyte apoptosis was greater in the medial (mean over four time points in DH: 2.9% ± 0.4, BS2: 3.1% ± 0.3) than lateral side (mean over four time points in DH: 1.8% ± 0.2, BS2: 2.4% ± 0.3) of AC and increased gradually as the disorder progressed in both strains (Table 1). The apoptotic chondrocytes were largely localised in the DZ of AC but also observed in the tidemark [22]. Between strains, the percentage of chondrocyte apoptosis was always higher in BS2 and statistically significant in the lateral side at 10 weeks (DH: 0.4% ± 0.1, BS2: 1.8% ± 0.3, *P* < 0.01). However, over time, only DH showed a significant progression of chondrocyte death by apoptosis in the medial side of AC between 24 and 30 weeks (*P* < 0.01).

The relationship between subchondral bone changes, chondrocyte apoptosis, and cartilage degradation in OA was investigated by comparing subchondral bone changes with cartilage degradation (as measured by OARSI score) and overall rate of chondrocyte apoptosis (Table 2). The caspase-3 expression in AC was significantly correlated with the microscopic score (*r* = 0.4, *P* < 0.01, *n* = 96; Spearman's partial correlation adjusted for age, strain, and weight). Of the 8 subchondral bone microstructural parameters considered only 1 (SbpTh) showed a weak but significant positive correlation with chondrocyte apoptosis (*r* = 0.2, *P* < 0.05, *n* = 96, partial Pearson correlation, adjusted for age, strain, and weight).

## 4. Discussion

**Figure 5:**
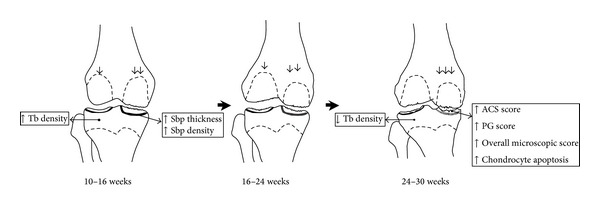
Temporal changes of AC and Sb during the progression of OA in DH. Between 10 and 16 weeks, a significant increase of Sbp thickness and density was observed in the medial side of tibial plateau, probably as a consequence of increasing loading on this side. Unlike the lateral side, Tb density did not substantially increase in the medial tibial epiphysis, this perhaps being due to the stress shielding effect as a result of medial Sbp sclerosis. As the disorder progressed, persistent loading on the medial side finally led to a progressive loss of chondrocytes, ACS integrity, and PG between 24 and 30 weeks of age. Within this time point, the lateral side showed a substantial decrease of Tb density. Arrows indicate shifting of load towards the medial side of the joint.

The present study reports longitudinal changes over 30 weeks in subchondral bone and cartilage in spontaneous animal models of OA. Our data show that changes in bone parameters occurred early in the disease course and that the pattern of subchondral bone change between the strains was different in the medial side, demonstrating an early increase followed by gradual decrease in some of the bone microstructural parameters in the OA prone DH strain. Cartilage degradation and subchondral bone changes were pronounced in the medial side of tibiae, and these progressed with age and correlated positively with chondrocyte apoptosis. In the medial condyles of DH animals* all* bone parameters except Tb.Pf tended to increase most between weeks 10 and 16. In contrast, measures of chondrocyte apoptosis and cartilage degradation increased most between weeks 24 and 30 suggesting that, in spontaneous guinea pigs models of OA progression occur first in bone and only later in the overlying cartilage. Sbp thickness and mineral density were the bone parameters that showed most pronounced and significant increases between 10 and 16 weeks. Moreover, increased chondrocyte apoptosis is preceded by Sbp thickening and was significantly associated with this process suggesting that apoptosis is likely to be a consequence of continuous remodelling in Sbp during the development of OA. These observations are depicted in Figure 5 and discussed below.

A high percentage of chondrocyte apoptosis was seen in the DZ and tidemark [22] and this is consistent with an earlier study by Adams et al. who noted a significant number of apoptotic chondrocytes in the calcified cartilage of Wistar rats and C57BL mice that also developed OA spontaneously [25]. Our findings are also supported by the results of a study using human osteoarthritic cartilage from the knee joint which found evidence of abundant vesicles (associated with early stages of apoptosis) budding out of the chondrocytes located in the DZ [26]. In the current study chondrocyte death was shown to be significantly positively associated with Sbp thickening and AC damage, and the progression of chondrocyte apoptosis was preceded by Sbp thickening. Thus, taken together, these observations suggest that chondrocyte apoptosis may be a consequence of Sbp changes during the development of OA, although we cannot rule out the involvement of this process in the initiation of OA since the BS2 also developed OA earlier than expected. Nevertheless, our data is consistent with the growing consensus that increased bone turnover, thickening of subchondral bone plate, and tidemark advancement all precede the progression of overlying articular cartilage deterioration [27].

We found that DH had a significant progression of chondrocyte apoptosis and cartilage damage in the medial side of AC (Figure 5). These changes were preceded by a marked increase in Sbp thickness and density between 10 and 16 weeks of age. Since DH and BS2 had a similar rate of growth and weight gain and none of the changes was seen in lateral side (the internal control for each strain) or in the medial side of BS2 within the same time points, the early changes of Sbp in the medial side of DH are interpreted as being most likely due to pathology rather than aging or growth. Moreover, the observed changes may also suggest that the Sbp plays an important role in the progression of AC damage and that and higher body weight could be a risk factor for an earlier onset of OA in DH. Previous studies of DH [1, 9, 10, 28] and other animal models of OA [11, 29] have also found an early progression of Sbp thickening. However, in some of these studies [1, 10], the initial SbpTh of OA animals was significantly lower than the control strains, thus suggesting that the thickening process is more important than the initial thickness of Sbp in the progression of OA [1].

The medial Sbp thickness in DH increased with age from 10 to 30 weeks but the BMD of Sbp decreased gradually from 16 weeks. The same was true for the Tb in the medial side, although the gradual decrease of BMD was not seen until 24 weeks. None of these changes were seen in the medial side of BS2. In human hip OA, the progressive thickening of Sb has been associated with hypomineralization, especially within regions closer to the joint surface [30]. Similarly, the Sbp and Tb thickening were also coincident with an increase of the Sb collagen: mineral ratio in DH [28], suggesting that the osteoarthritic Sb became sclerotic and yet undermineralized, probably as result of active Sb remodelling. Studies of animal models of OA have demonstrated that antiresorptive treatment (e.g., alendronate or calcitonin) following anterior cruciate ligament transection (ACLT) suppresses the bone resorption and AC degradation [2, 31, 32]. Furthermore, the resorptive agent also enhances the mineralization of newly formed bone alongside early bone formation and cartilage repair in rabbits with an osteochondral defect [33]. Although the efficacy of alendronate treatment for OA in DH was not conclusively established [34], the above findings suggest that the SbpTh is not the only determining factor for the acceleration of cartilage damage. Indeed, the thickening process that coincided with progressive Sb remodelling and hypomineralization could be an important event for the progression of cartilage degradation in OA.

Cartilage degradation and subchondral bone change progressed with age in both models of OA, in a manner consistent with findings from previous studies [1, 4, 10, 28, 35–39]. These changes were more pronounced in the medial than the lateral side of tibial epiphysis in both strains. A greater susceptibility of the medial side towards AC and Sb changes could be due to varus alignment following knee instability during the course of the disease. This hypothesis is supported by a study of subchondral bone changes in a canine ACLT-meniscectomy model which showed that the varus alignment leads to greater cartilage damage in the medial AC and subchondral bone loss in the lateral side of Tb [40]. Similarly, our spontaneous animal model of OA demonstrated a notable cartilage degradation in the medial side, whereas a decrease of Tb volume fraction, thickness, number, and connectivity in the lateral side of DH and BS2 between 24 and 30 weeks of age. Since the subchondral bone changes are largely influenced by loading [41], the loss of Tb in the lateral side (as indicated in the above studies) may further emphasize the likelihood of this region being unloaded as result of varus malalignment. The role of changes in bone loading driving OA is corroborated by the relationship between weight and pathology in the two models used in this study.

Shifting of the loading axis towards varus would impose extra load onto the medial side of the tibial epiphysis. This alteration would not only lead to medial AC degradation, but also induce bone formation in the same side. This hypothesis is supported by data from a study by Lindsey et al. who demonstrated an increase in Tb formation and AC degradation in the medial side of human tibia but loss Tb in the lateral side [42]. Moreover, Sharma et al. have confirmed that, either in varus or valgus human knee OA, there was bone formation in the diseased side whilst bone loss in the contralateral side of the tibia [43]. In our study, only the medial side of BS2 continued to increase in Tb formation until the end of study period. Moreover, despite having a lighter body weight, BS2 also had a slightly higher Tb number, density (measured by micro-CT), and connectivity than DH at 30 weeks of age. The heavier OA prone DH had more pronounced AC and Sb changes for the first 24 weeks, as expected. There may be a number of possible reasons for the differences in Tb changes at the final time point between the two strains. First, the increase of Tb connectivity in the medial side of BS2 could be a protective mechanism, slowing down the progression of OA in this strain. Second, the late Tb changes in BS2 may reflect the structural adaptation of subchondral bone in DH at the beginning of this study. Third, a significant thickening and stiffening of Sbp in the medial side of DH may reduce load and therefore the development of the underlying Tb due to a stress-shielding effect. Overall, the above findings highlight the importance of joint alignment in the structural adaptation of Sb to loading and progression of OA [44] and suggest a mechanism whereby ligament changes precede cartilage pathology in the DH model [45].

## 5. Conclusion

In summary, our data provide compelling evidence that chondrocyte apoptosis and cartilage degradation in osteoarthritis (OA) may be secondary to primary changes in the underlying bone in the two spontaneous guinea pig modes used in the study. Subchondral bone and cartilage changes were more pronounced in the medial side of tibial epiphysis (versus lateral side) and in DH (versus BS2). These findings are in agreement with the previous studies of DH and other animal models of OA. In the medial side of DH, the progression of Sbp thickening and stiffening preceded AC degradation and chondrocyte apoptosis suggesting that Sbp plays an important role in the early development of OA. The thickening of Sbp which coincided with hypomineralization at the later time points may reflect a continuous process of Sbp remodelling and contribute to the late progression of chondrocyte apoptosis and AC degradation in this spontaneous animal model of OA.

## Figures and Tables

**Table 1:** Body weight, microscopic scores, and percentage of caspase-3 of DH and BS2 at four time points.

	DH				BS2			
Age (weeks)	10	16	24	30	10	16	24	30
Body weight (g)	601.8 (5.9)^†^	845.0 (21.4)^∗†^	1083.3 (14.5)^∗†^	1071.7 (40.8)^†^	498.3 (13.6)^†^	729.2 (17.0)^∗†^	885.8 (14.5)^∗†^	902.5 (18.2)^†^
Microscopic score								
(i) Medial side	4.2 (1.0)	6.5 (0.9)	9.7 (1.9)	16 (0.8)^∗†^	3.2 (0.4)	5.8 (0.7)	9.0 (1.0)	12.5 (1.2)^†^
(ii) Lateral side	3.0 (0.4)	5.5 (0.8)	6.2 (0.8)	6.8 (0.9)	3.3 (0.6)	4.3 (0.3)	4.5 (0.4)	5.3 (0.4)
Caspase-3 (%)								
(i) Medial side	0.9 (0.3)	2.0 (0.2)	2.8 (0.5)	5.7 (0.6)*	1.5 (0.5)	3.0 (0.5)	3.1 (0.5)	4.8 (0.4)
(ii) Lateral side	0.4 (0.1)	1.6 (0.3)	2.5 (0.4)	2.6 (0.4)	1.8 (0.3)	2.2 (0.4)	2.3 (0.5)	3.2 (0.6)

Body weight of DH (*n* = 6) was significantly higher than BS2 (*n* = 6) at each time point (unpaired *t*-test, *P* < 0.01). Over time, both strains had a dramatic increase of body weight between 10 and 24 weeks (one-way ANOVA, *P* < 0.01). Total microscopic score is always higher in the medial side (vs. lateral side). In the medial side, the microscopic score of DH was significantly greater than BS2 at 30 weeks (Mann-Whitney *U* test, *P* < 0.05). Over time, only the medial side of DH showed a substantial increase of microscopic score and caspase-3 expression between 24 and 30 weeks (Kruskal-Wallis test, *P* < 0.01). The data are expressed as mean (SEM); ∗ and † indicate a significant difference between the adjacent time points and strains, respectively.

**Table 2:** Correlation between chondrocyte apoptosis and both cartilage degradation and Sb parameters.

	Caspase-3	
	*r*	*P*
Microscopic score	0.4	<0.01
BMD Sbp	0.2	<0.05
BMD Tb	0.1	<0.05
SbpTh	0.2	<0.05
BV/TV	0.1	>0.05
Tb.Th	0.1	>0.05
Tb.N	0.02	>0.05
Tb.Sp	0.1	>0.05
Tb.Pf	−0.02	>0.05
